# Defining the urine proteome in boys with posterior urethral valves: a pilot study

**DOI:** 10.3389/fcell.2026.1752740

**Published:** 2026-05-13

**Authors:** Xin Wang, Hanna Cortado, Liwen Zhang, Christina B. Ching, Ashley R. Jackson, John W. Froehlich, Richard S. Lee, Brian Becknell, Daryl McLeod

**Affiliations:** 1 Kidney and Urinary Tract Center, The Abigail Wexner Research Institute at Nationwide Children’s, Columbus, OH, United States; 2 Mass Spectrometry and Proteomics Facility, Comprehensive Cancer Center, The Ohio State University, Columbus, OH, United States; 3 Department of Urology, Nationwide Children’s Hospital, Columbus, OH, United States; 4 Division of Nephrology and Hypertension, Nationwide Children’s Hospital, Columbus, OH, United States; 5 Department of Urology, Boston Children’s Hospital, Boston, MA, United States; 6 Department of Surgery, Harvard Medical School, Boston, MA, United States

**Keywords:** chronic kidney disease, kidney and urinary tract injury, posterior urethral valves, single-cell RNA-seq, urine proteomics

## Abstract

**Introduction:**

Posterior urethral valves (PUV) is the most common cause of congenital bladder outlet obstruction and a major etiology of end-stage kidney disease in boys. There are significant knowledge gaps about the pathogenesis and prognostic indicators of kidney and bladder dysfunction in boys with PUV.

**Methods:**

We utilized liquid chromatography-tandem mass spectrometry to analyze the urine proteome in a pilot cohort of 20 boys with PUV compared to 20 unaffected controls, with a focus on estimated glomerular filtration rate (eGFR) variation within the PUV group and its impact on urine protein signatures. Eight complementary workflows for differential expression analysis were developed to ensure robust detection of differential protein expression. A single-cell RNA-seq atlas of 162,083 healthy kidney, ureter, and bladder cells was assembled to infer tissue and cellular origins of PUV-associated proteins.

**Results:**

In cases of PUV with preserved eGFR, upregulation of keratins and uroplakins was detected, suggestive of urothelial injury and remodeling that may reflect bladder dysfunction that occurs early in the disease process, even before a decline in renal function. In contrast, cases with low eGFR were associated with declining levels of the proteins related to viability and synthetic function of specific nephron and collecting duct segments, along with increased levels of proteins related to complement activation and inflammation, suggestive of progressive renal injury. The single-cell atlas provided biological context, identifying putative renal and urothelial cell sources for these proteins.

**Conclusion:**

This integrative analysis highlights biological pathways and proteins that may reflect kidney and urinary tract injury and dysfunction in a pilot cohort of boys with PUV. These initial findings warrant further testing in longitudinal studies with larger cohorts to link the urine proteome to clinically relevant kidney and bladder outcomes.

## 1 Introduction

Posterior urethral valves (PUV) occur in 1 in 4,000 live male births and represent a leading cause of pediatric chronic kidney disease (CKD) and end-stage kidney disease (ESKD) (Brownlee et al., 2019; van der Zanden et al., 2023; Benfield et al., 2003). PUV is defined by a partially obstructing membrane within the male prostatic urethra, which occurs early during fetal development, around 8 weeks post conception. Despite the singular anatomic defect associated with PUV, there is a wide spectrum of disease phenotypes that vary in severity depending on the onset and extent of bladder outlet obstruction (BOO). Close clinical surveillance is mandatory to identify early signs of CKD progression and intervene before permanent damage occurs (Shukla AaS, 2021).

The risk of CKD progression among boys with PUV is based on multiple factors, starting with the degree of fetal kidney damage and the resulting diminution in renal reserve at birth. Fetal “pop-off” mechanisms can decompress the urinary tract and a recent meta-analysis suggests they may serve a role in preserving renal function (Montero et al., 2024). Although there is no direct clinical measure of renal reserve, the serum nadir creatinine in the first year of life (SNC1) does provide guidance to identify children at risk for ESKD (McLeod et al., 2019). Unfortunately, SNC1 only measures the best kidney function in the first year and cannot predict future injury, which often occurs in PUV due to recurrent infections and pressure damage. Given the lifelong risk of additive insults to the urinary tract and kidney function in older children with PUV, an ideal set of biomarkers to improve risk stratification would be (Brownlee et al., 2019): specific to the unique ascending mechanism of injury (van der Zanden et al., 2023), allow serial measurements and (Benfield et al., 2003) provide early evidence of ongoing injury to allow time for clinical intervention aimed at mitigating CKD progression.

Given the unique mechanisms of kidney injury, PUV is especially amenable to unbiased approaches such as urinary proteomics to obtain pathological insights and unveil potential markers of CKD progression. The urine proteome consists of approximately 2,000–3,500 proteins in both healthy and diseased individuals, of which nearly 70% originate from the kidney and urinary tract (Swensen et al., 2021). Thus, the urinary proteome reflects local alterations in kidney and urinary tract health and can effectively function as a “liquid biopsy”, as shown in a variety of kidney and urinary tract disorders, including ureteropelvic junction obstruction (Chen et al., 2019; Mesrobian et al., 2010; Decramer et al., 2006; Froehlich et al., 2016), diabetic nephropathy (Limonte et al., 2022; Merchant et al., 2009), lupus nephritis (Zhang et al., 2008), and kidney allograft rejection (O'Riordan et al., 2004; Schaub et al., 2004; Sigdel et al., 2016). The unique and dynamic expression of such urinary proteins offers critical insights into organ and cell-specific functions, making them invaluable for biomarker discovery. Furthermore, among pregnancies complicated by PUV, studies of the fetal urine peptidome have identified those subjects who will experience ESKD in early childhood (Buffin-Meyer et al., 2020; Klein et al., 2013).

It is unknown, however, if urinary peptidomics or proteomics can predict CKD progression in the vast majority (∼90%) of children with PUV who do not reach ESKD in the first 1–2 years of life (McLeod et al., 2019). Thus, these published studies suggest an opportunity to apply urinary proteomics to older boys with PUV at varying stages of CKD. In the current pilot study, we aimed to determine if urinary protein signatures and corresponding biological pathways distinguish PUV versus healthy unaffected controls, as well as those cases of PUV with normal versus mild to moderately decreased eGFR. We integrated the urine proteome with single-cell RNA-seq data from the kidney, ureter, and bladder of normal subjects to infer putative cellular origins of these proteins. In doing so, we inferred organ- and cell-specific proteins that may reflect kidney and urinary tract injury and dysfunction in PUV.

## 2 Materials and methods

### 2.1 Human subject recruitment and sample processing

After obtaining Institutional Review Board approval (IRB18-00695) and informed consent/assent, urine samples were collected from 20 consecutive boys with clinically proven PUV (cases; Supplementary Table S1) and 20 age- and sex-matched controls (Supplementary Table S2). Cases were recruited from our multispecialty clinic. All cases were felt to be at their clinical baseline by their treating urologist and nephrologist, did not have signs or symptoms of illness, and had not experienced infections including UTI in the month preceding urine collection. Urine was collected between 9/2018 and 8/2021. Clinical details of the PUV cohort are provided in Supplementary Table S1. Healthy controls were recruited from the general pediatric clinic at the time of a well-child check or non-urologic complaint. Controls were screened to ensure they did not have active ill symptoms, history of illness in the past month, or known kidney or bladder anomalies. Urine was collected by volitional voiding or urine bag only. Urinalysis was completed via dipstick (Fisher Scientific, Pittsburgh, PA), and urine was stabilized in Assay Assure (Sierra Molecular, Incline Village NV) prior to processing. Samples were centrifuged at 4 °C at 750 × *g* for 8 min, followed by 3,000 rpm for 5 min to remove cellular debris. The supernatant and pellets were separated and stored at −80 °C, and analysis was restricted to a single freeze/thaw cycle.

### 2.2 Differential expression analyses, detection, and functional enrichments

Urine protein was extracted, digested with trypsin, and 200 ng of each sample was subject to liquid chromatography-tandem mass spectrometry. Details of sample preparation, protein quantification, and detection methods are described in the Supplementary Methods. To detect differentially expressed proteins, we utilized a comprehensive set of differential expression analysis (DEA) tools present in Bioconductor and designed for proteomic analyses (Zhu et al., 2020; Feng et al., 2023; Ahlmann-Eltze and Anders, 2019; Suomi et al., 2017; Choi et al., 2014; Smyth, 2005). For protein counts generated by Mascot, we mainly applied the Wilcoxon test for DEA detection (Supplementary Methods). For FragPipe intensity data, we applied seven statistical tools and pipelines to detect DEA: DEqMS (Zhu et al., 2020), DEP (Feng et al., 2023), proDA (Ahlmann-Eltze and Anders, 2019), ROTS (Suomi et al., 2017), MSstats (Choi et al., 2014), Limma (Smyth, 2005), and the Wilcoxon rank-sum test (Supplementary Methods). Protein intensities were normalized across samples using median normalization to correct for systematic variation in total signal intensity. To overcome missing values, we applied the k-nearest neighbors (Knn) using impute function from the MSnbase R package (Supplementary Methods). To gain insight into protein function enrichment, gene ontology (GO) analysis was performed for up- and downregulated proteins using the R package clusterProfiler v4.4.4 (Yu et al., 2012). A Chi-square test was used to test for statistically enriched pathways and FDR ≤ 0.05 was used as the significance threshold to detect enriched molecular functions, biological processes, and cellular components.

Cases with normal and low eGFR were identified based on serum creatinine and height measurements within 3 months of urine collection, using the modified bedside Schwartz equation (Schwartz et al., 2009). We detected differentially expressed proteins between controls and cases with normal eGFR (eGFR ≥ 90 mL/min/1.73 m^2^), as well as between cases with low eGFR (eGFR < 90 mL/min/1.73 m^2^) and normal eGFR. Differentially expressed proteins and GO enrichment terms were further analyzed, using the same strategy described above. Spearman correlation coefficients between MaxLFQ Intensity and eGFR values were calculated. Absolute Spearman correlation values (r) ≥ 0.7 or ≤ −0.7 identified proteins strongly associated with eGFR (Akoglu, 2018; Chan, 2003).

### 2.3 Single cell RNA-seq analyses

We collected published available single-cell or nucleus RNA-seq data from normal 13 human kidneys, 11 ureters, and 5 bladders via Gene Expression Omnibus (see Supplementary Methods). We analyzed single-cell datasets using Seurat, applying stringent quality-control, normalization, dimensionality reduction, clustering, and doublet detection. To account for variability across samples and technologies, we used Harmony for batch correction and dataset integration, followed by UMAP visualization and cell-type annotation. Differential expression and hierarchical clustering were then applied to characterize kidney, ureter, and bladder cell types and define organ- and cell type–specific expression patterns (see Supplementary Methods for details).

### 2.4 ELISA validation of PUV associated proteins

Commercial ELISA kits were utilized to quantify urine levels of APOA4 (Elabscience, Wuhan, China) and Clusterin (R&D Systems). The limit of detection was 2.81 ng/mL and 1.05 ng/mL for APOA4 and Clusterin, respectively. To take underlying differences in urine solute concentration into account, urine creatinine was quantified by colorimetric assay (Oxford Biomedical Research, Rochester Hills, MI). Each analyte was analyzed according to ng/mg urine creatinine as well as ng/mL urine.

### 2.5 Experimental design and statistical rationale

All statistical analyses were conducted using R software. Quantitative variables were expressed as mean ± standard deviation (SD) or median and interquartile range (IQR) for variables with non-normal distribution. The Shapiro-Wilk normality test was used to evaluate the normal distribution. Two-tailed Wilcoxon tests or other corresponding tests were employed to detect differential protein expression and differential gene expression in single-cell analyses. FDR or Bonferroni adjustments were applied for analyses where multiple hypothesis testing was applicable. Spearman correlations were employed to assess the similarity of cellular sources across organs within the kidney and urinary tract.

## 3 Results

### 3.1 Study overview

Proteomic profiling was performed by liquid chromatography-tandem mass spectrometry (LC/MS/MS) in urine samples collected from 40 boys, encompassing 20 cases with PUV and 20 unaffected, age-matched controls (Supplementary Tables S1, S2). Among PUV cases, 15 were diagnosed prenatally. The age at urine collection for proteomics was 6.8 years (IQR 6.0−10.2), which occurred 6.1 years after PUV ablation on average. The median eGFR was 88.5 ml/min/1.73 m^2^ (IQR 73.8−114.5). Ten patients with PUV had normal eGFR values (≥90 mL/min/1.73 m^2^). None of the cases had known genetic syndromes or associated congenital malformations. Two had urinary diversions at the time of urine collection, and one was performing clean intermittent catheterization. Other clinical features of the PUV cohort are summarized in Supplementary Table S1.

### 3.2 Multi-platform analysis identifies high-confidence differentially expressed proteins

**FIGURE 1:**
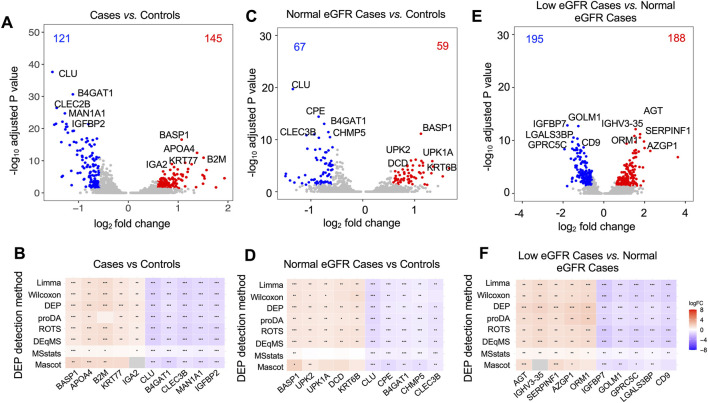
Urinary proteomic signatures distinguishing PUV cases by eGFR status and controls. Volcano plots illustrate differential protein expression between all PUV cases and controls **(A)**, PUV cases with normal eGFR and controls **(C)**, and PUV cases with low eGFR and controls **(E)**. The blue color shows the downregulated proteins, whereas red indicates the upregulated proteins. The labeled proteins are the top 5 upregulated proteins and 5 downregulated proteins ranked by fold change then adjusted P value. Robust detection of these labeled DEPs utilized eight distinct analytical workflows to distinguish PUV cases and controls **(B)**, PUV cases with normal eGFR and controls **(D)**, and PUV cases with low eGFR and normal eGFR **(F)**. The color scale represents the fold change, whereas the stars represent the significance levels. *p < 0.05, **p ≤ 0.01, ***p ≤ 0.001 detected by various DEP methods.

To enhance the accuracy and robustness of differentially expressed protein (DEP) detection, we implemented a comprehensive integrative approach using eight distinct analytical workflows (Supplementary Figure S1). This strategy uses diverse statistical modeling and sensitivity to data traits—like missing values, distribution, and variance—to enhance confidence in DEP identification through complementary methods strength. Using this integrative approach, we identified 266 DEPs (145 upregulated, 121 downregulated) when comparing cases to controls (Figure 1A). Among these, 89.47% of DEPs were consistently identified by at least two workflows, and 40.22% were detected by at least six workflows. The top five upregulated and downregulated proteins demonstrated consistent fold changes and statistical significance, with strong support from at least six analytical methods (Figure 1B). This strong cross-method concordance highlights the reproducibility of urinary proteomic signatures in PUV, enhancing confidence in the biological validity of downstream interpretations.

Among DEPs distinguishing cases compared to controls, the top five upregulated proteins were BASP1, APOA4, B2M, KRT77, and IGA2, while the top five downregulated proteins were CLU, B4GAT1, CLEC3B, MAN1A1, and IGFBP2 (Figures 1A,B; Supplementary Tables S3, S4). Differential expression of APOA4 and CLU was confirmed by ELISA, reinforcing the robustness of the proteomic findings (Supplementary Figure S2). Functional enrichment analysis revealed that upregulated proteins in cases were significantly associated with innate and humoral immune responses and keratinization (Supplementary Figure S3; Supplementary Table S5), including enrichment of innate immune effectors (S100A8, S100A9, CAMP, DEFA1, LYZ, LTF, DMBT1) and keratins (KRT6A, KRT6B, KRT77, KRT80) (Supplementary Figures S3C,D; Supplementary Table S5). Conversely, downregulated proteins in PUV were associated with wound healing, renal development, and epithelial tube morphogenesis (Supplementary Figure S3B; Supplementary Table S6), including AQP2, GPC3, COL4A1, and EGF (Supplementary Figures S3E,F; Supplementary Table S6).

### 3.3 Detection of proteins distinguishing controls, PUV cases with normal eGFR, and PUV cases with low eGFR

To determine the impact of PUV on the urinary proteome in the setting of normal glomerular function, we compared the proteomes of cases with normal eGFR versus controls (Figure 1C). The top 5 upregulated proteins were BASP1, UPK2, UPK1A, DCD, and KRT6B; and the top 5 downregulated proteins were CLU, CPE, B4GAT1, CHMP5, and CLEC3B (Figures 1C,D; Supplementary Tables S7, S8). Functional enrichment analysis revealed that these proteins are associated with biological processes such as keratinocyte differentiation, humoral immune response, wound healing, and tissue regeneration (Supplementary Figure S4A–D; Supplementary Tables S9, S10). Notably, PUV patients with normal eGFR exhibited a protein signature dominated by keratinocyte and urothelial markers (KRT1, KRT2, KRT5, KRT6A, KRT6B, KRT10, KRT14, KRT17, UPK2, UPK1A, S100P) (Supplementary Figures S3E, S4A).

To investigate the impact of reduced eGFR on the urinary proteome in the setting of PUV, we compared the proteomes of cases with low versus normal eGFR (Figures 1E,F). The top five upregulated proteins were AGT, IGHV3-35, SERPINF1, AZGP1, ORM1, whereas the top five downregulated proteins were IGFBP7, GOLM, GPRC5C, LGALS3BP, and CD9 (Figures 1E,F; Supplementary Tables S11, S12). This proteomic profile reflects activation of pathways associated with complement activation, coagulation, and fibrinolysis in cases with low eGFR, along with suppression of pathways associated with wound healing, keratinocyte differentiation, and kidney development (Supplementary Figure S5A–E; Supplementary Tables S13, S14).

**FIGURE 2:**
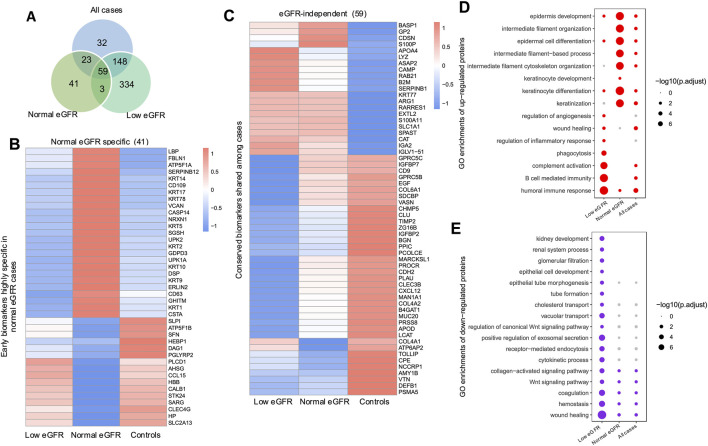
Detection of proteins distinguishing controls, PUV cases with normal eGFR and PUV cases with low eGFR. **(A)** Venn diagram illustrating the overlap of DEPs between the low eGFR, normal eGFR, and all case groups, in comparison to controls. **(B,C)** Heatmap displaying the expression profiles of DEPs associated with cases with normal eGFR **(B)**, and cases independent of eGFR **(C)**. The color scale represents the z-score of normalized MaxLFQ intensity for each protein. **(D)** Biological processes associated with upregulated proteins in each indicated group of cases compared to controls. **(E)** Biological processes associated with downregulated proteins in each indicated group of cases compared to controls.

To gain deeper insights regarding the specific effects of normal and low eGFR states on the PUV proteome, we identified DEPs that are either shared or unique to each group when compared to controls. Venn analysis identified 41 specific proteins in cases with normal eGFR; 334 in cases with low eGFR, and 59 in cases regardless of eGFR (Figure 2A). Among 41 specific proteins in cases with normal eGFR, multiple keratins—including basal urothelial cell markers (KRT5, KRT14, KRT17) and keratinocyte markers (KRT1, KRT2, KRT9, KRT10) — and uroplakins (UPK1A, UPK2) were elevated, suggesting urothelial injury and remodeling (Figure 2B). Consistent with this theme, GO enrichment analysis identified keratinocyte development and differentiation as specifically upregulated pathways (Figure 2D). Among 59 DEPs shared among all PUV cases regardless of eGFR, we identified reduced expression of tubular proteins including CLU, EGF, and DEFB1 (Figure 2C). Low eGFR cases exhibited downregulation of additional tubular proteins implicated in solute and water transport such as LRP2, CUBN, AQP1, AQP2, and UMOD (Supplementary Figure S6A,B). Corresponding GO enrichment analysis revealed significant repression of pathways related to kidney development in cases with low eGFR (Figure 2E).

### 3.4 Detection of proteins associated with eGFR in cases

**FIGURE 3:**
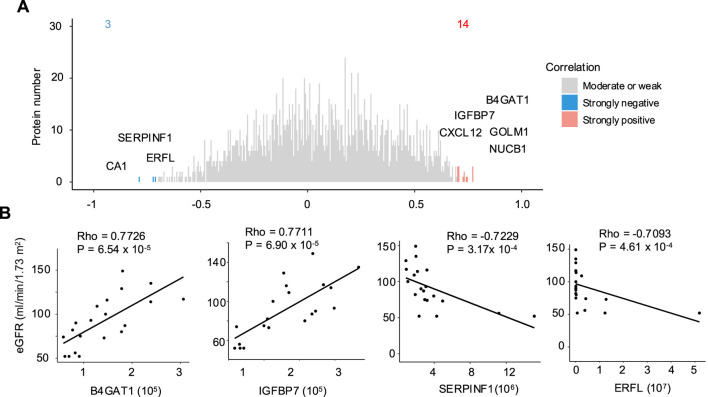
Detection of proteins strongly correlated with eGFR levels in cases. **(A)** The correlation density distributions of urinary proteins based on Spearman’s Rho. Urinary proteins were evaluated by normalized MaxLFQ intensity. Three categories were defined based on Rho values: strongly negative (≤ −0.7) (blue), strongly positive (≥0.7) (red) and moderate or weak (> −0.7 and <0.7) (grey). The labeled proteins are those with the strongest positive and negative correlations with eGFR based on absolute Rho values. **(B)** Examples show the positive and negative correlations between eGFR and protein expression for B4GAT1, IGFBP7, SERPINF1 and ERFL.

Next, we investigated the strength and direction of association between urinary protein expression and eGFR. 14 proteins exhibited a positive correlation (Spearman’s Rho coefficient ≥ 0.7), while 3 proteins displayed a negative correlation (Rho ≤ −0.7) (Figure 3A). The top 5 positively correlated proteins were B4GAT1, IGFBP7, GOLM1, CXCL12, NUCB1, whereas the negatively correlated proteins were CA1, SERPINF1, and ERFL (Figures 3A,B; Supplementary Tables S15, S16).

### 3.5 Assembling a comprehensive single-cell atlas of the healthy human kidney and urinary tract

**FIGURE 4:**
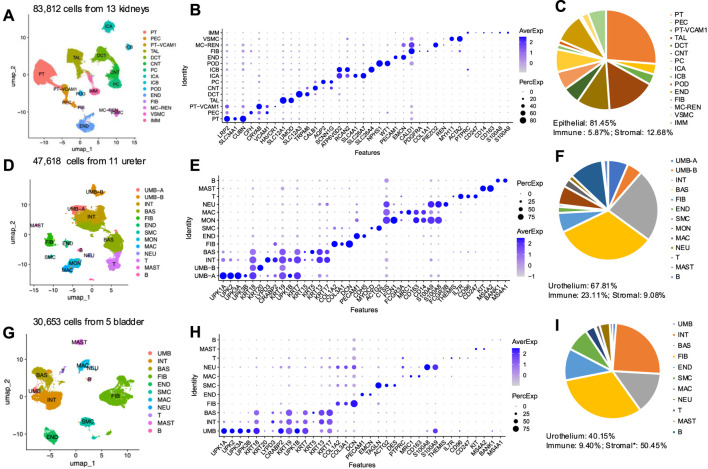
Integration of single-cell transcriptomes from normal human kidney, ureter, and bladder. **(A)** UMAP of 83,812 cells collected from 13 healthy kidneys. PT, proximal tubule cells; PEC, Parietal epithelial cells; PT-VCAM1, proximal tubule with VCAM1 positive cells; TAL, thick ascending limb of the loop of Henle; DCT, distal convoluted tubule; CNT, connecting tubule; POD, Podocyte; PC, principal cells; ICA, type A intercalated cells; ICB, type B intercalated cells; FIB, fibroblast; MC-REN: smooth muscle with REN positive; VSMC, vascular smooth muscle cells; IMM, immune cells. **(B)** Dot plot showing average published available marker gene expression and proportion expressed for each cell type. **(C)** Pie chart showing relative abundance and percentage of cell types. **(D)** UMAP of 47,618 cells from 11 healthy ureters. UMB-A, umbrella cell cluster A; UMB-B umbrella cell cluster B; INT, intermediate cells; BAS, basal cells; END, endothelium cells; SMC, smooth muscle cells; MON, monocyte cells; MAC, macrophage; NEU, neutrophil; T, T cells; MAST, mast cells; B, B cells. **(E)** Dot plot showing average published available marker gene expression and proportion expressed for each ureter cell type. **(F)** Pie chart showing relative abundance and percentage of ureter cell types. **(G)** UMAP of 30,653 cells from 5 healthy bladders. **(H)** Dot plot showing average published available marker gene expression and proportion expressed for each bladder cell type. **(I)** Pie chart showing relative abundance and percentage of bladder cell types.

To infer putative cellular sources of DEPs, we constructed a single-cell (sc)RNA-seq atlas using archival data from healthy human kidneys, ureters and bladders (Supplementary Table S17). ScRNA-seq data from 279,840 single cells were collected. After stringent quality control, doublet elimination, and batch effect removal, 162,083 high-quality single cells were used for integration, clustering, and visualization. These included 83,812 kidney cells (Figure 4A), 47,618 ureter cells (Figure 4D), and 30,653 bladder cells (Figure 4G). We identified 15 major canonical cell types in the kidney (Figures 4A–C), 13 in the ureter (Figures 4D–F), and 11 in the bladder (Figures 4G–I), strongly supported by established available markers (Figures 4B,E,H). Within these healthy organs, we observed varying presence of immune cells, comprising 5.87% of the kidney, 23.11% of the ureter, and 9.40% of the bladder cell populations (Figures 4C,F,I). Cross-organ analyses revealed a high degree of similarity among immune cells, endothelial cells, smooth muscle, and fibroblasts across the kidney, ureter, and bladder, whereas epithelial cells displayed organ-specific variability across the urinary tract (Supplementary Figures S7A–C).

### 3.6 Inferring the putative cellular and organ sources of PUV-associated proteins

**FIGURE 5:**
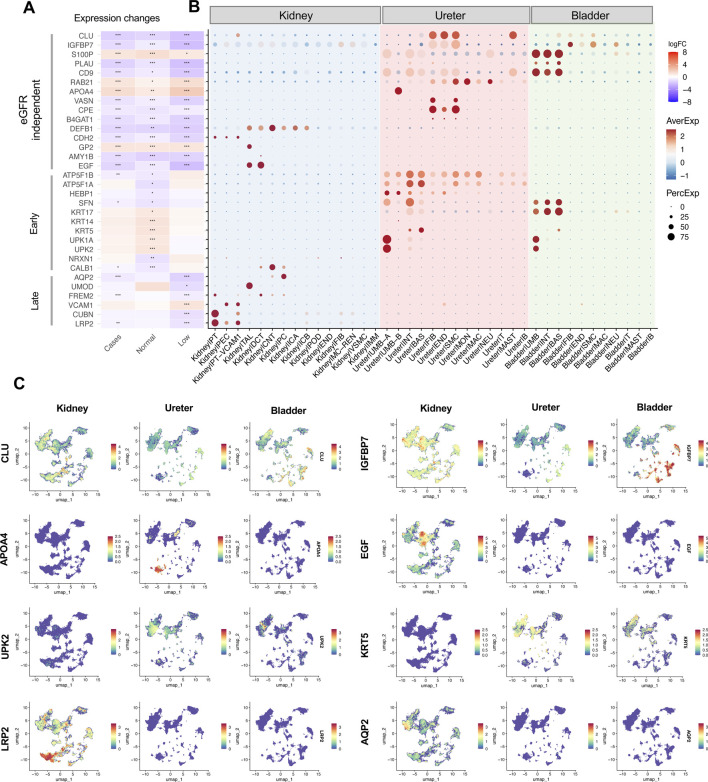
Inferring cellular sources of DEPs. **(A)** Heatmap showing fold change in each group comparison: cases vs. controls, normal eGFR cases vs. controls, and low eGFR cases vs. controls. The color scale represents fold change, while asterisks indicate significance levels based on adjusted p-values (*p < 0.05, **p ≤ 0.01, ***p ≤ 0.001) for conserved PUV associated proteins (DEPs that distinguished cases with PUV from controls regardless of eGFR), early PUV associated proteins (DEPs specifically enriched in PUV cases with normal eGFR), and late PUV associated proteins (DEPs specifically enriched in cases with low eGFR). Some early or late PUV associated proteins appear significant across multiple groups but are not considered DEPs due to low fold changes. **(B)** Dot plots showing expression levels of corresponding genes across different cell types in the kidney, ureter, and bladder. The color scale represents the average expression level of each gene within a given cell type, with deeper color intensity indicating high expression. The dot size reflects the proportion of cells within that cell type expressing the genes, with larger dots indicating a higher percentage of expressing cells. **(C)** Representative examples of expression distribution highlight PUV associated proteins enriched in specific tissues.

To infer the cellular origins of PUV-associated proteins, we leveraged the scRNA-seq atlas from healthy kidney and urinary tract organs. Hierarchical clustering of 59 DEPs that distinguished PUV cases from controls irrespective of eGFR revealed seven distinct protein clusters with organ- or cell-specific expression profiles (Supplementary Figures S8A,B). Cluster 1 contained proteins (such as LYZ, ARG1, and SERPINB1) that are typically expressed by ureteral immune cells, whereas proteins expressed in clusters 6 and 7 (such as CXCL12, CLEC3B, and CPE) are expressed by ureteral stromal cells (Figures 5A–C; Supplementary Figures S8A,B). We identified increased APOA4 in PUV, which was specifically enriched in umbrella cells from ureteral urothelium (Figures 5A–C; Supplementary Figures S8A,B). Additionally, we detected PUV downregulated proteins in cluster 5 that are highly enriched in the kidney and expressed by distinct cell types in the nephron and collecting duct, including DEFB1, EGF, CDH2, GP2, and AMY1B (Figures 5A–C; Supplementary Figures S8A,B).

Among 41 DEPs that distinguished PUV cases with normal eGFR from controls, we identified enriched expression of their corresponding transcripts in the ureter and bladder rather than in the kidney (Supplementary Figure S9). These DEPs are particularly enriched in keratin and uroplakin proteins. Accordingly, transcripts encoding KRT5, UPK2, UPK1A, KRT14, KRT17, KRT10, and KRT80 are highly expressed in the ureter and bladder urothelium (Figures 5A–C; Supplementary Figure S9). Certain DEPs derive from transcripts that are more widely expressed in healthy ureter than bladder and kidney, such as ATP5F1A, ATP5F1B, HEBP1, and GHITM, and may therefore reflect ureter-specific DEP in PUV cases. Conversely, certain DEPs with reduced expression in PUV cases with normal eGFR are encoded by transcripts with cell-type-specific expression patterns in the kidney, such as CALB1, SLC2A13, and NRXN1 (Figures 5A–C; Supplementary Figure S9). Similarly, interrogation of scRNA-seq data suggested that the 334 DEPs in cases with low eGFR derive from distinct clusters of transcripts with organ- and cell-type- specific expression profiles (Supplementary Figure S10). Further analysis identified increased levels of VCAM1 and decreased urine levels of tubular proteins, including AQP2, UMOD, CUBN, and LRP2, that map to specific nephron and collecting duct cell populations (Figures 5A–C; Supplementary Figure S10).

## 4 Discussion

PUV represents the most common cause of congenital bladder outlet obstruction and a leading cause of ESKD in boys. Two factors contribute to CKD progression in boys with PUV (Brownlee et al., 2019): Reduced renal reserve at birth from impaired fetal nephrogenesis and (van der Zanden et al., 2023) Acquired kidney injury, often due to hostile bladder dynamics and urinary tract infections that occur despite PUV ablation. The convergence of these factors results in 15–20 percent progression to ESKD during childhood (Sarhan et al., 2008; DeFoor et al., 2008; McLeod et al., 2019; Robinson et al., 2024; Garne et al., 2025). In this exploratory pilot study, we analyzed the urinary proteome to gain insights regarding DEP and potential underlying biological mechanisms of kidney and urinary tract injury in PUV. In cases with preserved eGFR, we observed increased levels of keratins and uroplakins, linked to pathways involved in urothelial injury and remodeling. Conversely, cases with reduced eGFR showed decreased levels of proteins essential for nephron and collecting duct cell viability and function, alongside higher levels of proteins related to complement activation and inflammation. Integrating scRNA-seq data with the urinary proteome identified candidate cellular and organ-specific sources of DEPs, providing important context for biological processes associated with PUV. Our study positions urinary proteomics as a powerful approach to investigate the pathogenesis and progression of CKD among patients with PUV.

### 4.1 Comprehensive scRNA-seq reference of the normal kidney and urinary tract

We present the first comprehensive scRNA-seq reference of the normal human kidney and urinary tract. Our single-cell analysis shows diverse urothelial, stromal, and immune cells in the ureter and bladder, with more immune cell diversity than the kidney. This underscores the need for comparative studies of their epithelial and immune microenvironments. The high immune cell presence in the ureter suggests a role in local immune surveillance, that may be crucial for urinary tract health and detecting bacteria from vesicoureteral reflux or impaired bladder emptying in PUV. Likewise, the diversity of urothelial cells in the ureter may enable it to respond uniquely to changes in urine volume or pressure in children with PUV. An important caveat of this approach is that cellular sources of urinary proteins were inferred based on scRNA-seq results from normal subjects without PUV. Future studies will benefit from efforts to localize PUV-associated proteins to their predicted cellular origins in biopsy or surgical specimens from subjects with PUV. Further exploration of these differences might reveal insights into how adaptation and injury occur, as well as identify potential therapeutic targets for urinary tract disorders like PUV.

### 4.2 Conserved differentially expressed proteins in PUV cases

Among DEPs that distinguish PUV from controls, we identified increased expression of proteins responsible for the innate immune response (LYZ, ARG1, CAMP) that mapped to immune cell populations such as neutrophils, monocytes, and macrophages. We showed PUV cases had higher urine β2‐microglobulin, possibly indicating increased proximal tubular reabsorption, linked to CKD progression in fetal and postnatal PUV cohorts (Heikkilä et al., 2021; Dreux et al., 2018). We also detected increased APOA4 levels in PUV, which is predominantly expressed in normal ureteral urothelium, as indicated by scRNA-seq. Serum APOA4 levels have been associated with renal impairment in diabetic nephropathy (Cheng et al., 2018) and mutations in *APOA4* have been linked to tubulointerstitial kidney disease (Kmochová et al., 2024). A recent study identified a relationship between increasing urine APOA4 levels and higher susceptibility for chronic renal allograft deterioration (Kee et al., 2025). While glomerular filtration of circulating APOA4 might contribute to higher urinary APOA4 in PUV, our findings suggest the ureteral urothelium could also be a source of APOA4. Additionally, we found that certain PUV downregulated proteins–including DEFB1, EGF, CDH2, GP2, and AMY1B—are primarily expressed in the kidney, often in a cell-specific manner. In particular, urine EGF levels map to the loop of Henle and distal convoluted tubule and are associated with tubular viability and synthetic function of these nephron segments, and reduced levels predict CKD progression (Ono et al., 2024; Menez et al., 2021; Harskamp et al., 2023; Wu et al., 2020). Likewise, reduced levels of proteins like DEFB1, CDH2, GP2, and AMY1B may also reflect injury and loss of reserve in their respective nephron and collecting duct segments and cells of origin. Together, these findings highlight the activation of multiple kidney injury pathways in PUV.

### 4.3 PUV-associated proteins in cases with normal eGFR

We paid particular attention to DEPs between PUV cases with normal eGFR and controls, with the mindset that DEPs may indicate mechanisms of early parenchymal injury as well as protective adaptations in this cohort. The predominant biological pathways in PUV cases with normal eGFR are associated with intermediate filament generation and keratinocyte differentiation, due to the upregulation of numerous cytokeratins. We hypothesize that these DEPs, along with increased expression of Uroplakin 2 (UPK2) and UPK1A, reflect alterations in the urothelial lining of the urinary tract–a cell population that is rich in KRT and UPK expression. Consistent with this concept, scRNA-seq identified bladder and ureteral urothelium as candidate cellular sources of these proteins. Interestingly, preclinical models of urinary tract obstruction have shown that renal urothelium undergoes injury-induced remodeling, marked by increased expression of KRTs and UPKs, which assemble an apical plaque that promotes urothelial compliance and impermeability, thereby mitigating kidney injury (Girshovich et al., 2012; Jackson et al., 2019; Jackson et al., 2018; Jackson et al., 2024; Carpenter and McHugh, 2017; Bec et al., 2013). Despite normal eGFR, these patients may manifest preclinical signs of renal injury, as evidenced by decreased levels of the distal nephron marker CALB1. Reduced urinary CALB1 has been reported in humans with the distal nephron segment injury (Iida et al., 2014), supporting this observation. These findings encourage future research to identify cellular sources—possibly via single-cell transcriptomics of urinary sediment in PUV patients—and monitor urine urothelial proteins (e.g., KRTs and UPKs) over time, linking changes to bladder, kidney pathology, and treatments.

### 4.4 PUV-associated proteins in cases with low eGFR

Our proteome analysis identified numerous proteins with altered expression in individuals with low eGFR, encoded by transcripts that are abundant in the healthy kidney, ureter, and bladder, indicating potential injury or dysfunction in these urinary tract parts. Notably, urine angiotensinogen (AGT) levels were elevated in cases with low eGFR. Similar increases in urine AGT have been reported in children with ureteropelvic junction obstruction (Taranta‐Janusz et al., 2013), type 1 diabetes mellitus and autosomal dominant polycystic kidney disease (Ba Aqeel et al., 2019; Park et al., 2020), where levels predict subsequent eGFR decline. Urine AGT levels are reflective of the intrarenal renin-angiotensin system (RAS), which is implicated in the etiology of CKD progression in obstructive nephropathy (Chevalier, 1999). We also detected increased urinary VCAM levels in PUV cases with low eGFR and identified a renal parietal cell and a subset of proximal tubular cells as potential sources of VCAM. Urinary VCAM levels are linked to CKD progression. Research shows VCAM+ proximal tubular cells that fail to repair and attract leukocytes, leading to inflammation and fibrosis (Kirita et al., 2020; Melchinger et al., 2024; Wu et al., 2024; Reck et al., 2025). Emerging studies suggest that VCAM drives epithelial-immune crosstalk, which can be targeted to slow CKD progression in preclinical models (Melchinger et al., 2024; Reck et al., 2025). Certain downregulated proteins in cases with low eGFR exhibit cell-specific expression within renal tubular epithelial cells, as suggested by scRNA-seq analysis, including CUBN and LRP2 (proximal tubules), UMOD (loop of Henle), and AQP2 (principal cells). The reduced proteins in PUV urine suggest impaired kidney function or segment loss. Decreased UMOD indicates limited synthesis and viability in the nephron’s thick ascending limb, which is linked to CKD progression (Limonte et al., 2025; Melchinger et al., 2022). A previous study identified reduced urine AQP2 levels in boys with PUV compared to age-matched controls (Trnka et al., 2012). AQP2 serves an essential role in water reclamation, and its reduced expression may account in part for the limited urine concentrating capacity in boys with PUV. Further studies are warranted to investigate the relationship between these urine proteins and tubular function. These findings were also supported by the identification of downregulated pathways in cases with low eGFR related to glomerular filtration, kidney development, and epithelial tube morphogenesis - indicating potential early nephron injury.

### 4.5 Study limitations

This was a pilot, exploratory study in a small, clinically heterogeneous group of boys with history of PUV and age-matched controls. The limited number and clinical heterogeneity characteristics of urine samples from cases with PUV constrained the statistical power, and further studies in a separate, larger PUV cohort are required to determine the generalizability of our findings. On average, the urine proteome was investigated 6.1 years following PUV ablation (median 6.4 years; Supplementary Table S1). Consequently, we cannot comment on the influence of prenatal features, such as fetal pop-off mechanisms, on the urine proteome. Kidney function in PUV cases was assessed solely on the basis of Cr-based eGFR within 3 months of urine collection. While these boys with PUV were felt by their treating physicians to be at their clinical baseline, it is conceivable that changes in eGFR occurred in the time between Cr measurement and urine collection. Moreover, bladder urodynamics were not performed within this 3 month time frame, preventing us from associating DEP with bladder dysfunction. The small sample size prevented us from determining the impact of urological management, such as clean intermittent catheterization or urinary diversion, on the urine proteome. Likewise, we were unable to assess the impact of complications of PUV, such as recurrent UTI, on the urine proteome. Moreover, this study lacked longitudinal assessment of the proteome and clinical course of each PUV case over time, thus preventing the evaluation of the stability of these DEPs and their longitudinal clinical associations. Additionally, the use of single-cell data from healthy controls limits the confirmation of these urinary protein origins in PUV. Finally, most cases of PUV had normal or only mildly reduced renal dysfunction, such that the full spectrum of CKD progression in PUV was incompletely represented. Larger prospective studies with detailed phenotyping of bladder function, management strategies, and longitudinal kidney outcomes will be necessary to confirm these associations and establish their translational relevance.

## 5 Conclusion

Our study identified DEPs and biological pathways that illuminate potential mechanisms of parenchymal injury in PUV. Integrating proteomics with single-cell analysis, we inferred cellular origins of PUV associated proteins in the kidney and urinary tract. Further research is needed to track these DEPs over time in more patients, explore their sources, links to interventions and outcomes, and the roles of their biological pathways in obstructive uropathy.

## Data Availability

All datasets supporting the conclusions of this article are included within the article and its additional files. The proteomic raw datasets can be accessed by MassIVE through accession number MSV000099270 or PRIDE through the accession number PXD068784. All the single-cell datasets were collected from the Gene Expression Omnibus (GEO). Human normal kidney: GSM4008619, GSM4008620, GSM4008621, GSM4008622, GSM5837792, GSM3823939, GSM3823940, GSM3823941, GSM4572192, GSM4572193, GSM4572194, GSM4572195, and GSM4572196. Human normal ureter: GSM5578032, GSM5578033, GSM5578034, GSM5578035, GSM5578036, GSM5578037, GSM5578038, GSM5578039, GSM5578040, GSM5578041 and GSM4008665; human normal bladder: GSM3723357, GSM3723358, GSM3723359, GSM3980126, and GSM3980127. Human single cell analyses followed standard workflow and we have now deposited the source code at GitHub: https://github.com/gucascau/HumanUrinaryTractSystem.git. The optimized proteomic analyses are now deposited at: https://github.com/gucascau/PUVUrinaryProteomic.git.

## Supplementary material

SUPPLEMENTARY FIGURE S1 Optimized workflows for differential expression analysis of urine proteome data. Raw protein quantification was performed using FragPipe and Mascot. Data were normalized using multiple approaches (quantile, VSN, median, or none) and missing values were imputed with k-nearest neighbors (Knn). DEP analyses were carried out using multiple statistical pipelines (DEP, DEqMS, Limma, Wilcoxon, MSstats, proDA, and ROTS). Final DEP integration was performed using multiple strategies, including Fisher’s method, Stouffer’s Z-score, Tippett’s method, and permutation-based empirical integration, with Fisher’s method selected for the final results.

SUPPLEMENTARY FIGURE S2 ELISA confirmation of DEP in PUV cases vs. Controls. APOA4 and CLU urine levels were measured by commercial ELISA and expressed as ng/mL urine or ng/mg Creatinine (Cr). P-values are shown above each plot (Mann-Whitney U test).

SUPPLEMENTARY FIGURE S3 Biological process enrichment and protein-level associations in PUV cases vs. controls. GO analysis indicates the top ten biological processes associated with cases **(A)** and controls **(B)**. **(C)** The cnetplot illustrates the associations between upregulated proteins and enriched biological processes, including humoral immune response and keratinization. **(D)** The upregulated proteins shown in **(C)** were identified across multiple proteomic pipelines. **(E)** The cnetplot illustrates the associations between downregulated proteins and enriched biological processes, including wound healing, renal system development, and epithelial tube morphogenesis. **(F)** The downregulated proteins shown in **(E)** were identified across multiple proteomic pipelines. In **(C,E)**, the color scale represents protein fold change between cases and controls. Dot size indicates the number of detected proteins involved in each biological process. In **(D,F)**, the color scale indicates the fold changes, while stars denote statistical significance levels detected by various DEP methods: p < 0.05 (*), p ≤ 0.01 (**), and p ≤ 0.001 (***).

SUPPLEMENTARY FIGURE S4 Biological process enrichment and protein-level associations in PUV cases with normal eGFR vs. controls. GO analysis identified the top ten biological processes associated with proteins activated **(A)** and repressed **(B)** in normal eGFR cases vs. controls. **(C)** The cnetplot illustrates the associations between upregulated proteins and enriched biological processes, including keratinocyte differentiation and humoral immune response. **(D)** The cnetplot illustrates the associations between downregulated proteins and enriched biological processes, including wound healing and regeneration. **(E)** Representative DEPs are associated with keratinocyte and urothelial markers, the immune response, and collagen production. In **(C,D)**, the color scale represents protein fold change between normal eGFR cases and controls. Dot size indicates the number of detected proteins involved in each biological process. In **(E)**, the color scale indicates the fold changes, while stars denote statistical significance levels detected by various DEP methods: p < 0.05 (*), p ≤ 0.01 (**), and p ≤ 0.001 (***).

SUPPLEMENTARY FIGURE S5 Biological process enrichment and protein-level associations in PUV cases with low eGFR compared to those with normal eGFR. GO analysis indicates the top ten biological processes associated with proteins activated **(A)** and repressed **(B)** in low eGFR cases. **(C)** The cnetplot illustrates the associations between upregulated proteins and enriched biological processes, including complement activation, coagulation, and fibrinolysis. **(D)** The cnetplot illustrates the associations between downregulated proteins and enriched biological processes, including wound healing and kidney development. **(E)** Representative DEPs are involved in complement activation, coagulation, wound healing, keratinocyte differentiation, and kidney development.

SUPPLEMENTARY FIGURE S6 Proteins specifically expressed in PUV cases with low eGFR. **(A)** Heatmap displaying the expression profiles of DEPs identified in cases with low eGFR. **(B)** Heatmap displaying DEPs associated with kidney development that are altered in cases with low eGFR. The color scale represents the z-score of normalized MaxLFQ intensity for each protein.

SUPPLEMENTARY FIGURE S7 The cellular comparisons between the kidney, ureter, and bladder. **(A)** UMAP of 162,083 integrated high-quality single cells from the kidney, ureter, and bladder. **(B)** Integrated UMAPs for the kidney, ureter, and bladder. **(C)** Correlation analyses were performed across defined cell types in the kidney, ureter, and bladder. Spearman correlation was used to assess gene expression similarities across each cell type.

SUPPLEMENTARY FIGURE S8 Tracing the cellular origins of urinary proteins that distinguish cases and controls. **(A)** Hierarchical clustering of DEPs that distinguished PUV cases from controls, irrespective of eGFR, reveals seven distinct protein clusters, each enriched for specific tissue or cell-type expression profiles. Dot plots indicate putative cellular sources of these DEPs based on the healthy scRNA-seq atlas within the kidney, ureter, and bladder. The color scale represents the average expression level of each gene within a given cell type, with deeper color intensity indicating high expression. The dot size reflects the proportion of cells within that cell type expressing the genes, with larger dots indicating a higher percentage of expressing cells. **(B)** Heatmap showing the fold change in urinary levels of each DEP when compared to healthy controls. The order of proteins corresponds to the hierarchical clustering of DEPs from **(A)**. The color scale reflects log_2_ fold change values, with red indicating upregulation and blue indicating downregulation. Statistical significance is indicated by asterisks (*p < 0.05, **p ≤ 0.01, ***p ≤ 0.001; adjusted p-values).

SUPPLEMENTARY FIGURE S9 Tracing the cellular origins of DEP between cases with normal eGFR and controls. **(A)** Hierarchical clustering of DEPs that distinguished PUV cases with normal eGFR from controls revealed five distinct protein clusters, each enriched for specific tissue or cell-type expression profiles. Dot plots indicate putative cellular sources of these DEPs based on the healthy scRNA-seq atlas of the kidney, ureter, and bladder. The color scale represents the average expression level of each gene within a given cell type, with deeper color intensity indicating high expression. The dot size reflects the proportion of cells within that cell type expressing the genes, with larger dots indicating a higher percentage of expressing cells. **(B)** Heatmap illustrating fold change in urinary levels of each DEP when compared to healthy controls. The order of proteins corresponds to the hierarchical clustering of DEPs from **(A)**. The color scale represents log2 fold changes, with statistical significance indicated by asterisks (*p < 0.05, **p ≤ 0.01, ***p ≤ 0.001; adjusted p-values).

SUPPLEMENTARY FIGURE S10 Tracing the cellular origins of DEP between cases with low eGFR and controls. **(A)** Hierarchical clustering of DEPs that distinguished PUV cases with low eGFR from controls revealed distinct protein clusters, each enriched for specific tissue or cell-type expression profiles. Dot plots indicate putative cellular sources of these DEPs based on the healthy scRNA-seq atlas of the kidney, ureter, and bladder. The color scale represents the average expression level of each gene within a given cell type, with deeper color intensity indicating high expression. The dot size reflects the proportion of cells within that cell type expressing the genes, with larger dots indicating a higher percentage of expressing cells. **(B)** Heatmap illustrating fold changes in urinary levels of each DEP when compared to healthy controls. The color scale represents log2 fold change, with statistical significance indicated by asterisks (*p < 0.05, **p ≤ 0.01, ***p ≤ 0.001; adjusted p-values).
