# Supplementary figures and images for: Defining the urine proteome in boys with posterior urethral valves: a pilot study
Source: Front Cell Dev Biol. 2026 May 13;14:1752740. doi: 10.3389/fcell.2026.1752740 (PMC13212451; doi:10.3389/fcell.2026.1752740)

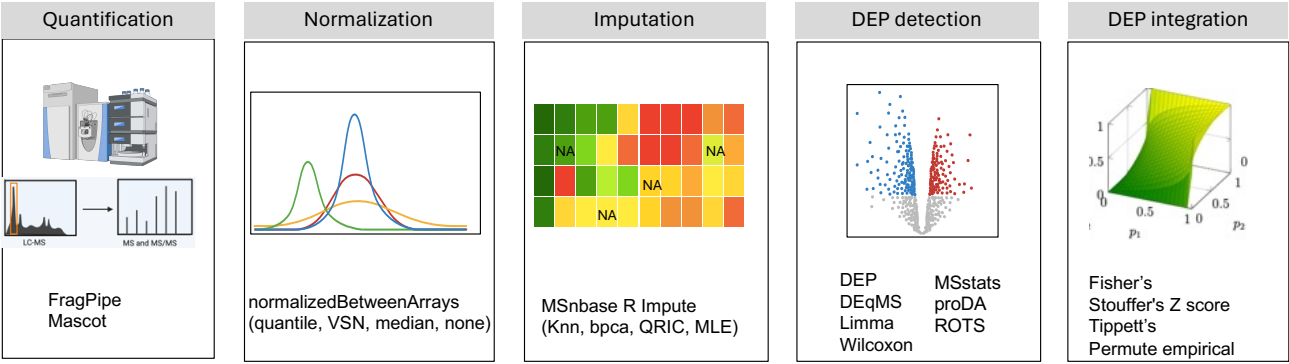

Figure S1

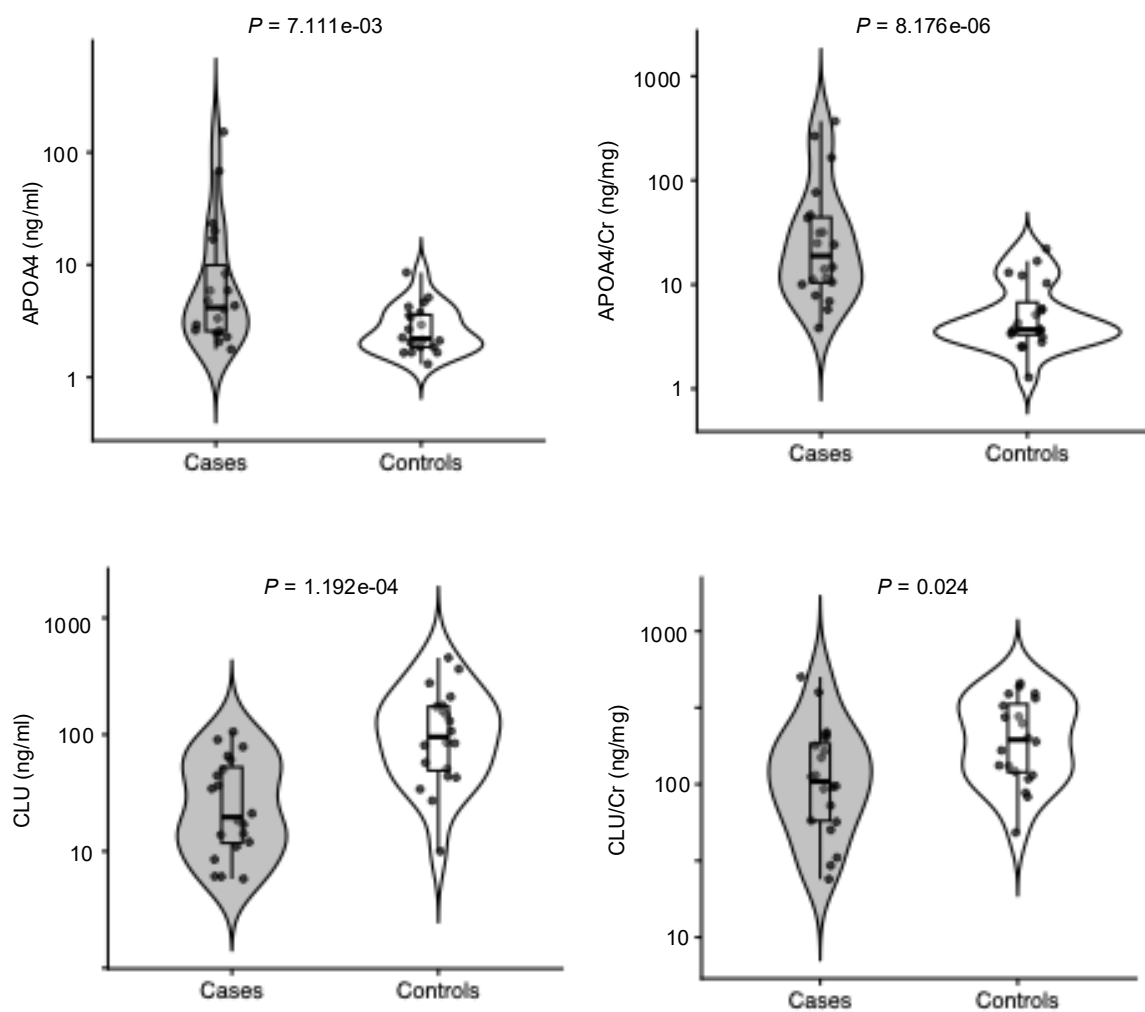

Figure S2

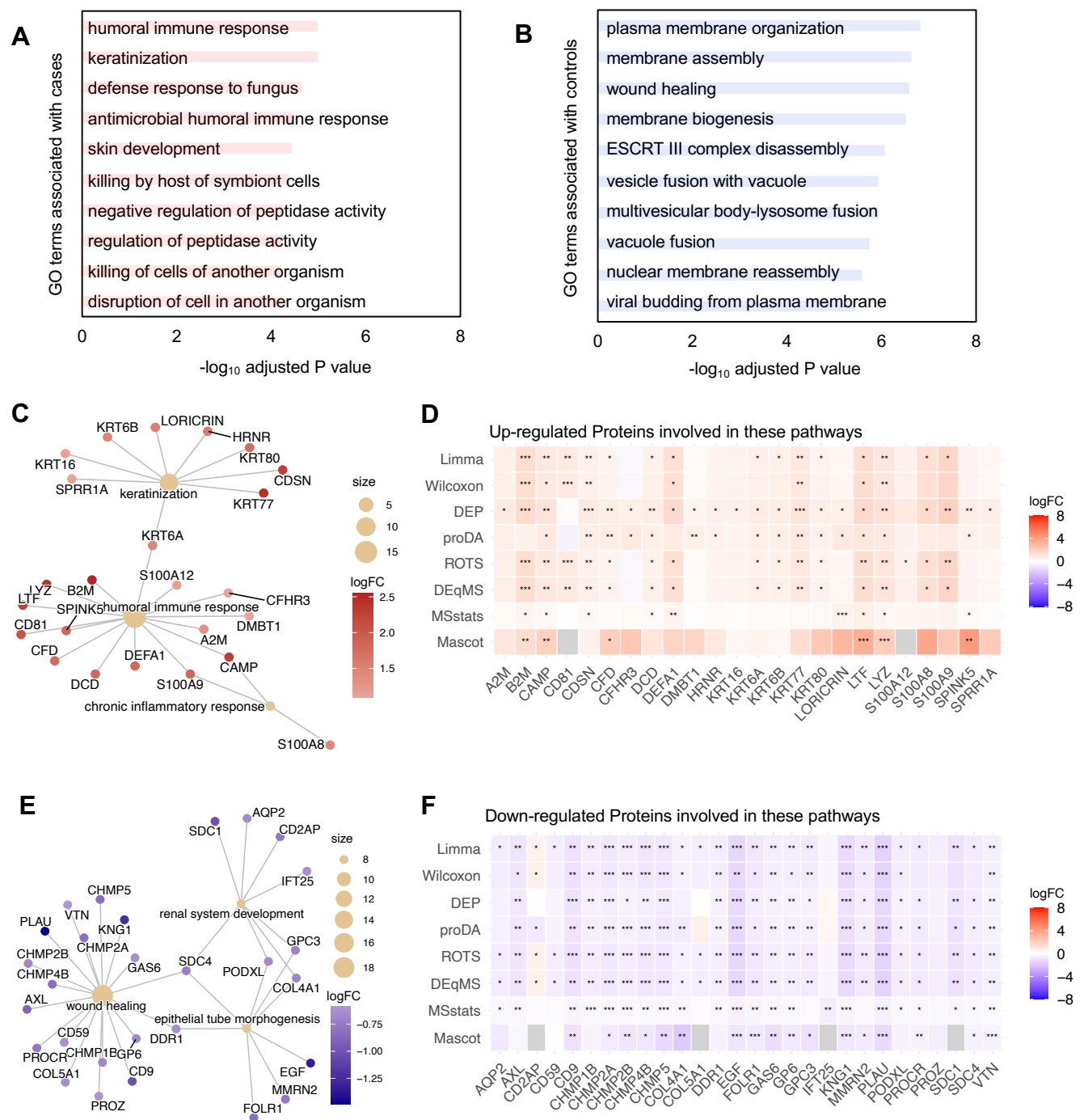

Figure S3

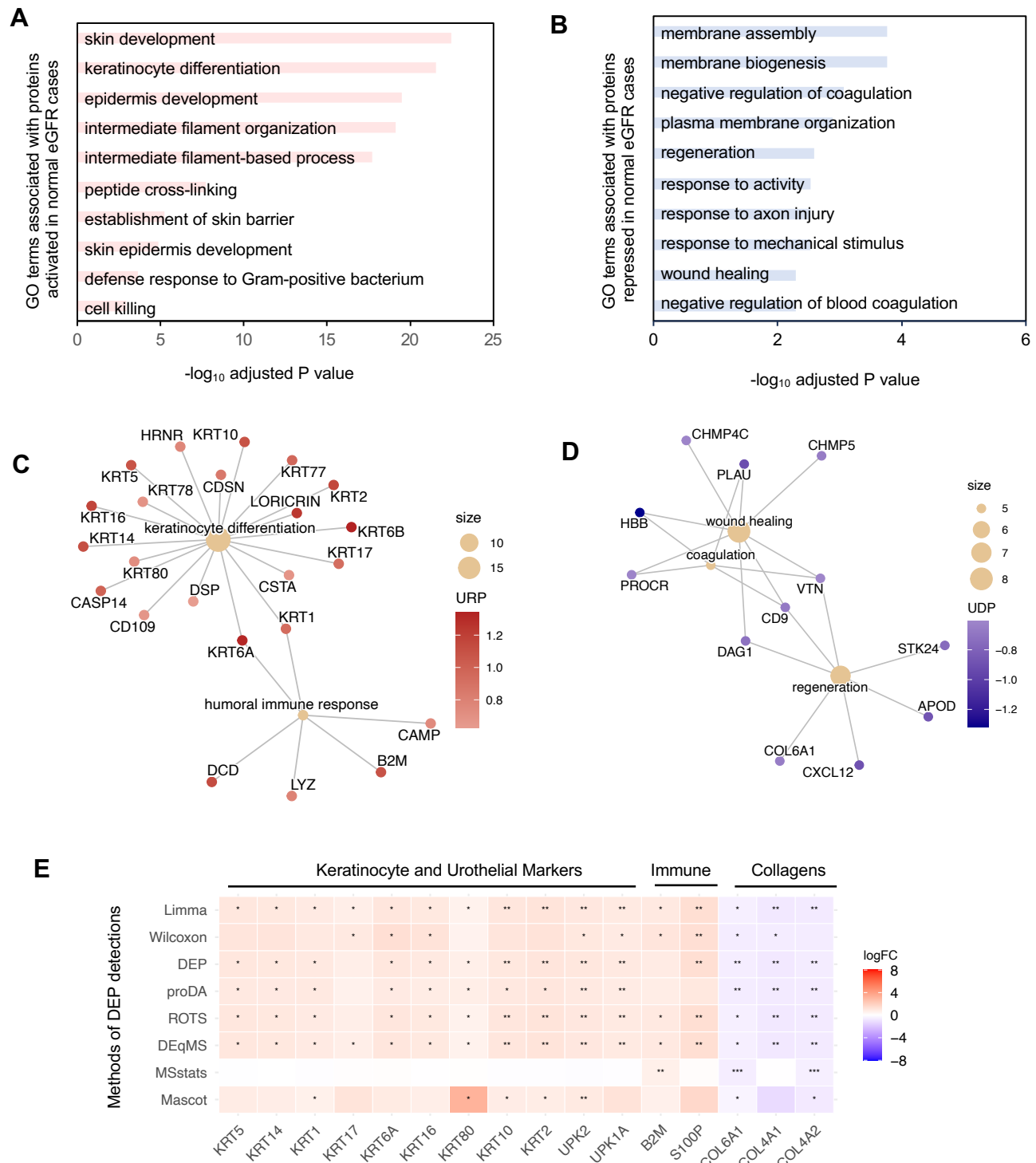

Figure S4

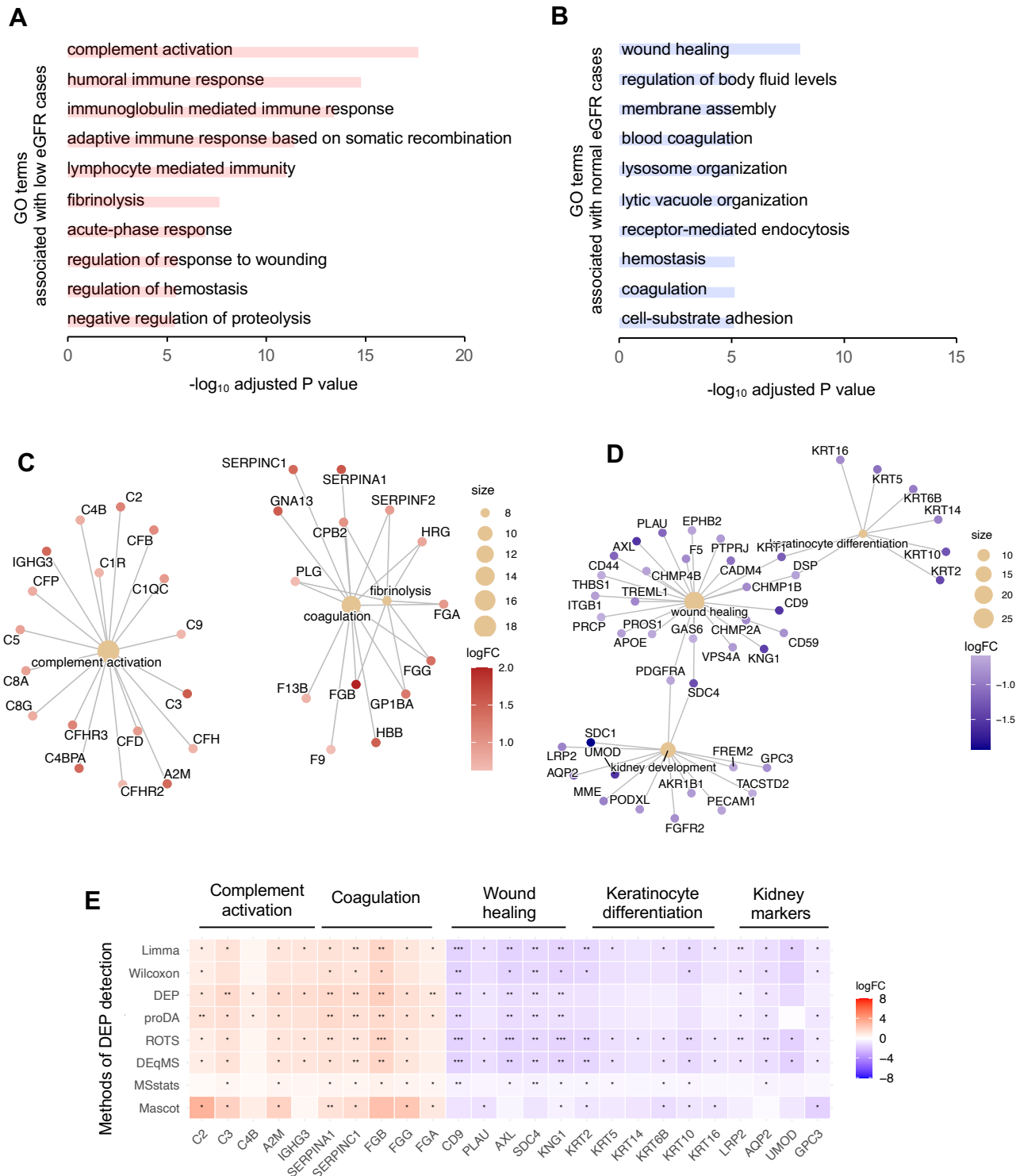

Figure S5

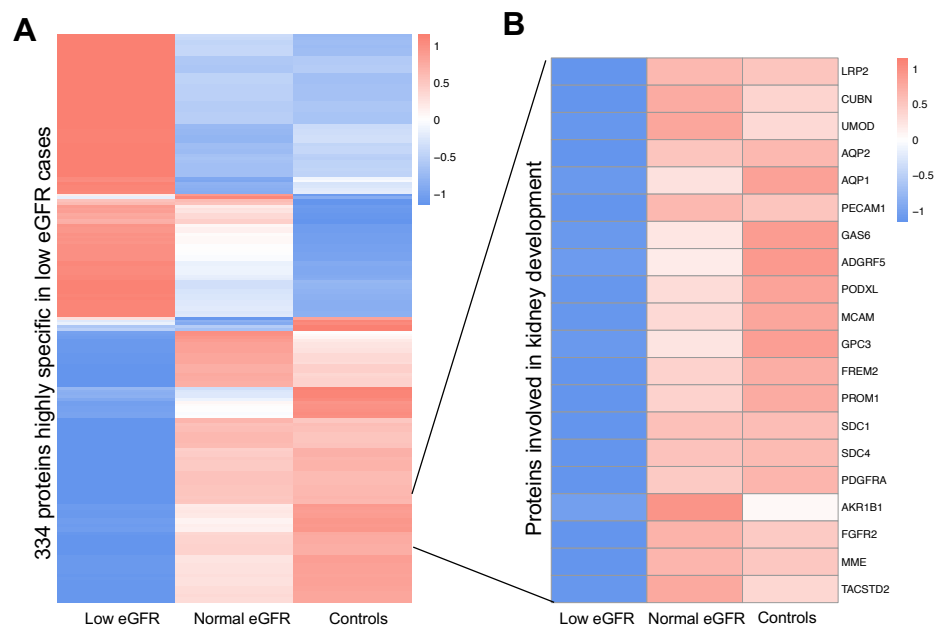

Figure S6

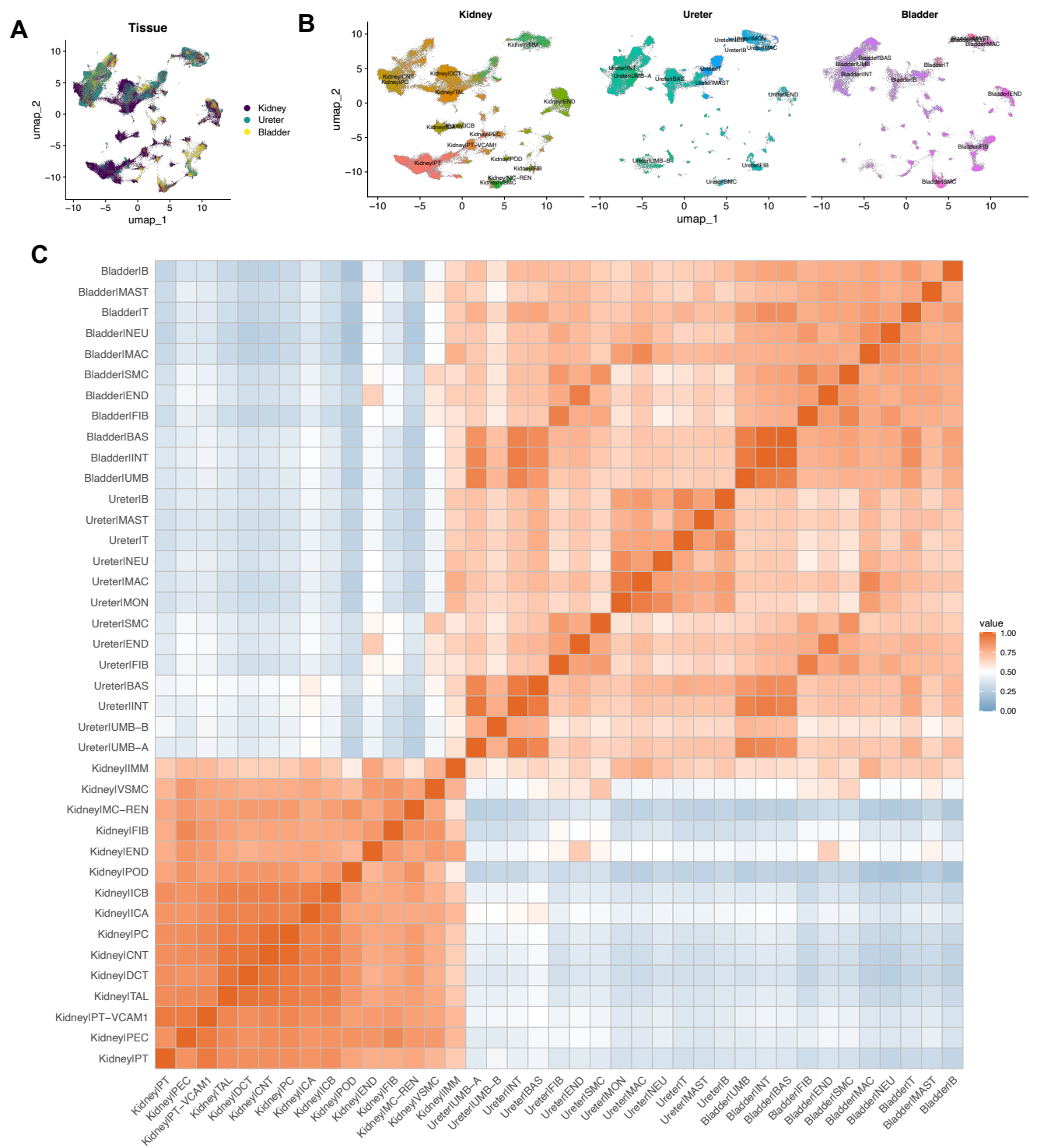

Figure S7

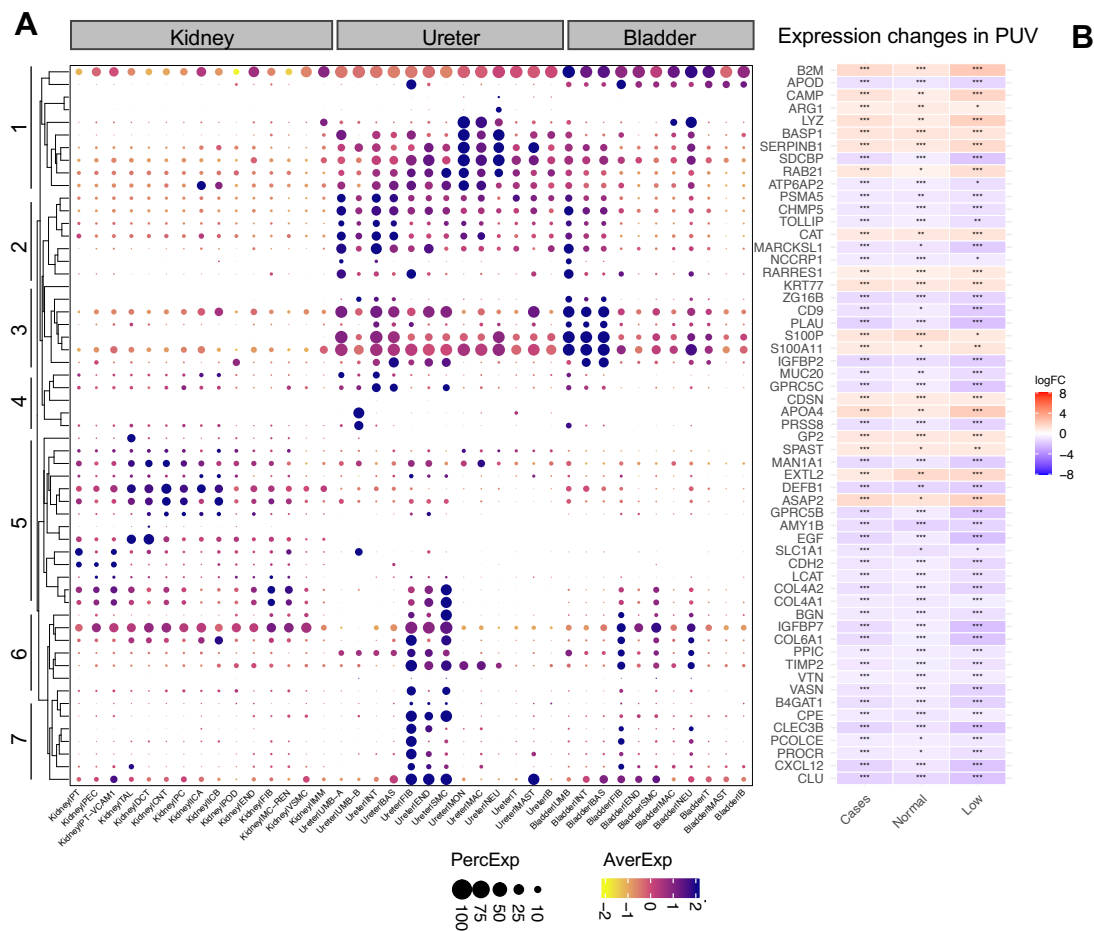

Figure S8

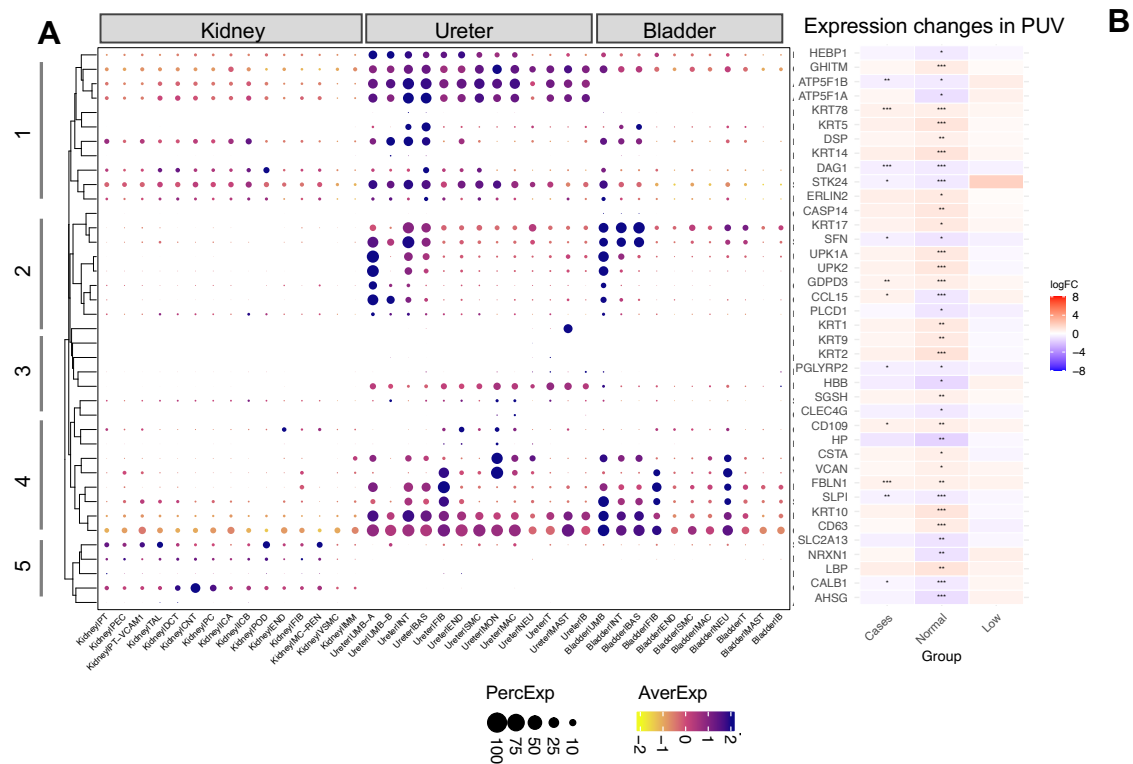

Figure S9

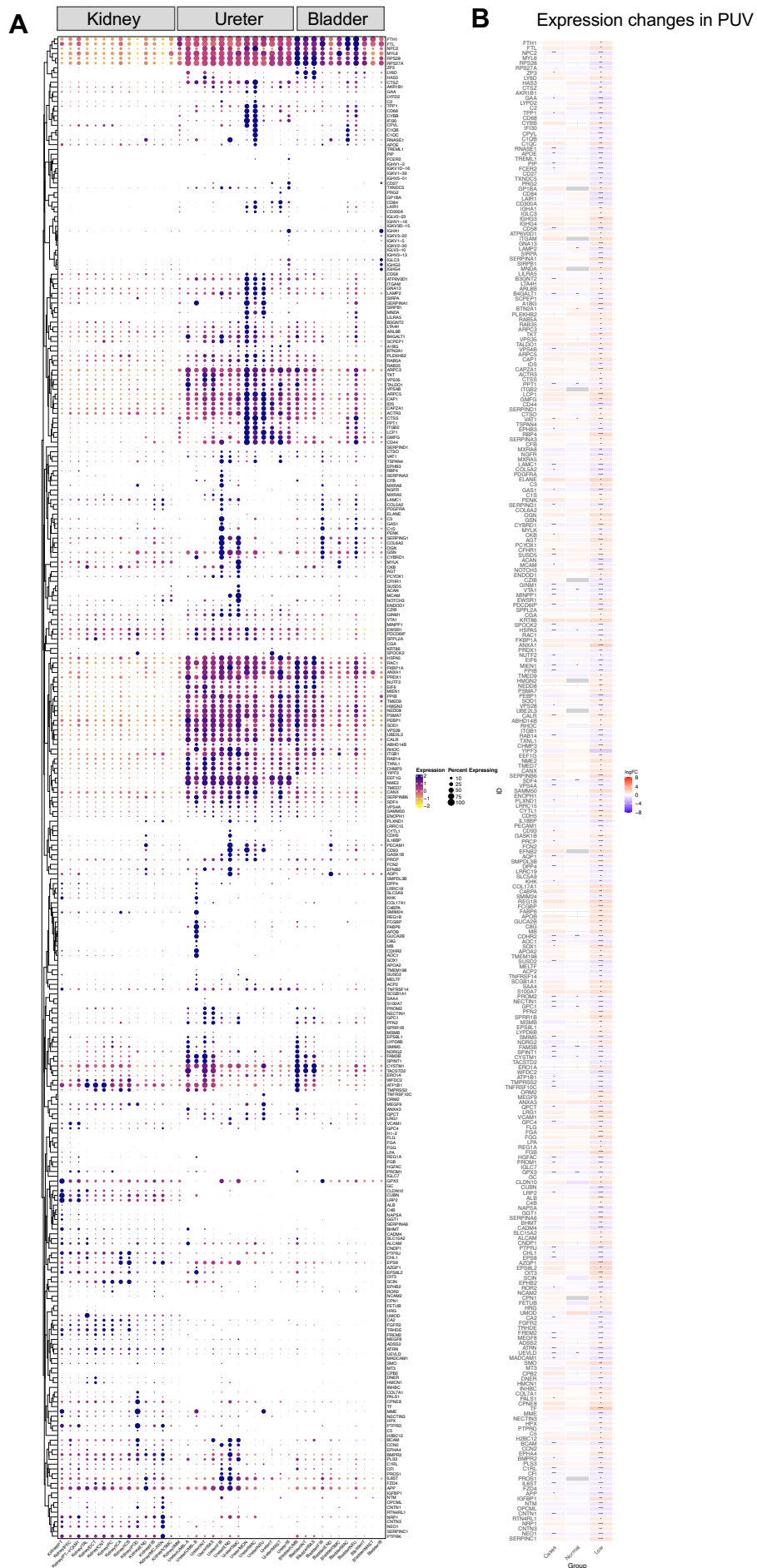

Supplement: Supplementary file 1 [file DataSheet1.pdf]
