# Supplementary material for: Defining the urine proteome in boys with posterior urethral valves: a pilot study
Source: Front Cell Dev Biol. 2026 May 13;14:1752740. doi: 10.3389/fcell.2026.1752740 (PMC13212451; doi:10.3389/fcell.2026.1752740)
Supplement: Supplementary file 3 [file DataSheet2.docx]

**Supplementary Material**

**Sample preparation and protein quantification.** Proteins were precipitated by mixing 400 μL of urine with 1600 μL of ice cold 100% acetone. Samples were washed with acetone, air dried, and resuspended in 5% SDS in 50 mM triethylammonium bicarbonate (TEAB). Proteins were prepared for trypsin digestion using S-Trap technology (ProtiFi, Fairport, NY). Next, 70 μg of protein was digested overnight at 37°C using sequencing-grade trypsin dissolved in 50 mM TEAB. Peptides were eluted using 40 μL of 50mM TEAB, 0.2% formic acid (FA), and 0.2% FA in acetonitrile (1:1 v/v), sequentially. The eluates were pooled together, dried in a vacuum centrifuge, and resuspended in 20 µL of 0.1% FA. Peptide concentration was determined by absorbance at 280 nm using a Nanodrop instrument (Thermo Fisher Scientific, Waltham, MA).

**Protein detection and label-free quantification.** Proteins were identified by nano-liquid chromatography-nanospray tandem mass spectrometry (LC/MS/MS) on a timsTOF Pro mass spectrometer (Bruker, Billerica, MA) equipped with a CaptiveSpray source operated in positive ion mode. Samples were separated on a C18 reverse phase column using a Bruker nanoElute UHPLC system. 200 ng of peptide was injected for each analysis. Two methods were used for protein and peptide detection. The first method employed Mascot Daemon (Matrix Science version 2.7.0, Boston, MA) to search against the Uniprot Human database. A decoy database was also searched to determine the false discovery rate (FDR), and peptides were filtered according to the FDR. The significance threshold was set at p < 0.05. Valid proteins were identified based on <1% FDR and detection of at least two peptides. The second method was using FragPipe(1-3) (v22.0) with its default settings for label-free data-dependent acquisition for quantification. Proteomic data from the 20 case and 20 control samples were searched against the Uniprot Human Reviewed 2025_01 Release (20,418 entries) using the LFQ-MBR workflow within FragPipe. Contaminants and decoys were added by FragPipe. PSM validation was performed by Percolator(4), protein inference by ProteinProphet (5), and FDR filtering by Philosopher(6) within the FragPipe workflow. IonQuant(7) was performed with intensity normalization and Match between runs (MBR), with default settings. FragPipe generated various types of expression matrices at both the peptide and protein levels, including total protein counts, unique spectral counts, and intensities, MaxLFQ intensities calculated using the MaxLFQ algorithm.

**Differential expression analysis based on protein counts detected by Mascot Daemon**

Proteomic counts generated by Mascot were processed by data filtering and normalization. In total, 2,123 urinary proteins from 20 cases and 20 controls were detected from the LC-MS/MS analyses. To overcome missing values, we filtered out any protein that was sparsely quantified in samples, i.e., a total count less than five across all samples. Then, quantile normalization method was used to eliminate or minimize technical variability. Differential expression analysis was conducted to identify proteins that were differentially expressed between cases and controls. The Wilcoxon rank-sum test was used to assess differences in protein levels between two groups. P values were adjusted for multiple testing using the FDR approach. Fold changes (FC) were calculated as = mean (ΔC _patients_) − mean (ΔC _controls_) and converted on a log_2_ scale.

**Optimizing differentially expressed proteins by various proteomic workflows.**

Given the presence of missing values and low expression of protein counts, we implemented a comprehensive optimization strategy for the DEP detection, incorporating intensities-based quantification along with spectral and peptide counts. Three key preprocess steps were applied to protein MaxLFQ intensities: filtration, normalization, and imputation, were conducted. For filtration, proteins not annotated as “Homo sapiens” (e.g. Ovis arises and Sus crofa in our datasets) were removed, along with proteins intensity not detected in at least 90% of the total samples. For normalization, these MaxLFQ intensities values were log-transformed and then quantile-normalized using the normalizedBetweenArrays from Limma R package. For missing value imputation, we applied the k-nearest neighbors (Knn) using impute function from the MSnbase R package. To further test whether these normalized intensities are normally distributed, we further applied Shapiro-Wilk normality test across all samples, indicating not normalized data but with biomodal distribution of these log-transferred and normalized intensities. Therefore, we will not consider parametric test that assume normalization, e.g., T-test and ANOVA.

To detect the differentially expressed protein, we took a comprehensive set of published available DEA tools that downloaded from Bioconductor and specifically designed for proteomic analyses. The analyses were performed using MaxLFQ intensities obtained from FragPipe.

The following statistical tools and pipelines were employed:

1. DEqMS (Differential Expression analysis with q value and MS-specific weights): Following preprocessing, differential expression analysis was performed using the DeqMS pipeline, which incorporate peptide-level quantification into variance modelling. Initially, linear modeling was conducted using limma package, and the resulting model were further refined by integrating peptide counts to improve the variance estimation. Protein abundance was summarized by aggregating peptide-spectrum match (PSM) quantification into protein quant in each sample. The spectraCountBayes function was used to perform empirical Bayes variance shrinkage to enhance sensitivity and specificity in protein-level inference based on the median number of unique peptides across samples.
2. ROTs (Reproducibility optimized test statistic). Using the preprocessed MaxLFQ intensities, we used ROTS package to identify robust DEPs. We configured the number of bootstrapping (B) and the number of top-ranked features for reproducibility optimization (K) to 10000 and 500 respectively to optimizes the reproducibility of top-ranked features in group-preserving bootstrap. This bootstrap approach will maximize the consistency of DEP detection with stable signal across repeated sampling.
3. DEP (Differential Enrichment analysis of Proteomics data): We followed the DEP pipeline designed for robust and reproducible analysis of mass spectrometry proteomics data for differential protein expression or differential enrichment. We used the raw MaxLFQ intensities and applied the functions in DEGs for variance normalization (vsn), imputation (knn) and statistical testing of differentially expressed proteins.
4. proDA (Probabilistic Dropout Analysis): proDA was used to analyze label-free MS data without imputing missing values. Instead, it models missingness with a sigmoidal dropout function fit for each sample. This probabilistic model is then used to estimate protein means and their uncertainty. Differential abundance was assessed using the Wald test implemented in the test_diff function.
5. MSstats: We used MSstats.csv generated from Fragpipe for the analyses in MSstats. Log transformation, normalization (equalizeMedian), feature selection (all) and missing value imputation were applied to preprocess the MaxLFQ values using default parameters of dataProcess. Differential changes in protein abundance across conditions was tested using a linear mixed-effects model.
6. Additional statistical Tests: To complement the above analyses, we applied the Wilcoxon rank-sum test as a non-parametric method to detect differences between conditions, as well as the Limma linear modeling framework (limma::lmFit and eBayes) to perform empirical Bayes-moderated t-tests. Wilcoxon rank-sum test and Limma were applied directly to preprocessed MaxLFQ intensities (filtering, normalization, and imputation). All p-values were adjusted for multiple testing using the Benjamini–Hochberg method.

To evaluate the impact of normalization and imputations, we also run the alternative pipelines. For normalization, we tested approaches without normalization and other normalization methods, such as “center.median”, “vsn”. For imputation, we also compared methods such as “bpca”, “QRIC” and “MLE” methods from MSnbase.

**Integration of seven methods of DEPs**

Differential expression analysis was integrated using these eight complementary statistical pipelines: Limma, DEqMS, DEP, proDA, ROTS, Wilcoxon, MSstats and Wilcoxon-Mascot. A protein was considered differentially expressed based on both absolute Log2FC (1.5-fold change) and adjusted p value (<=0.05). For the integration of log2FC, we chose the log2FC having the biggest absolute value among all the sub-log2FC (excluding Wilcoxon-Mascot). To integrate statistical significance across methods, we applied Fisher’s combined probability test to aggregate p-values.

$$X^{2}= -2 \sum_{i=1}^{k} ln(pi)$$

Specifically, for $k$ independent test with p values p_1_, p_2_, …p_k_. This test statistics followed a chi-squared distribution with 2$k$ degrees of freedom under the null hypothesis. The resulting combined p-values were then adjusted for multiple hypothesis testing using the Benjamini–Hochberg false discovery rate (FDR) correction. Additionally, we also provided three other different P value integration methods, including, Tippett, Stouffer’s Z-score and Permutation empirical.

**Human kidney and urinary tract single cell collections.** We collected published available single cell or nucleus RNA-seq data from normal human kidney, ureter, and bladder via Gene expression Omnibus (GEO). For the normal kidneys, we downloaded 13 single-cell datasets GSM4008619, GSM4008620, GSM4008621, GSM4008622, GSM5837792, GSM3823939, GSM3823940, GSM3823941, GSM4572192, GSM4572193, GSM4572194, GSM4572195, and GSM4572196, spanning three studies (GSE134355(8), GSE131882(9, 10), GSE151302(10, 11)). These libraries contributed 184,280 raw single kidney cells. For the normal ureter, we gathered 11 single-cell datasets GSM5578032, GSM5578033, GSM5578034, GSM5578035, GSM5578036, GSM5578037, GSM5578038, GSM5578039, GSM5578040, GSM5578041 and GSM4008665, spanning two studies (GSE184111(12) and GSE134355(8)). These libraries included 62,065 raw single ureter cells. For the normal bladder, 5 single cell datasets GSM3723357, GSM3723358, GSM3723359, GSM3980126, and GSM3980127, spanning two studies (GSE134355(8) and GSE129845(13)) were collected. These libraries generated 33,495 raw single bladder cells.

**Single cell RNA-seq analyses.** The single cell analyses were primarily conducted using Seurat v5.1.0(14). The raw datasets in each organ were filtered for low-quality cells using QC thresholds based on distribution assessments. Low-quality cells were defined as those having ≥ 30% mitochondrial gene counts, fewer than 100 or more than 8,000 gene counts, or total RNA counts greater than 16,000. Following this filtering step, the standard Seurat workflow was applied to each sample. Briefly, global-scaling normalization was used to ensure comparable expression levels across all cells. Next, variable feature selection was performed, and the data were scaled accordingly. Principal component analysis (PCA) was conducted to reduce data dimensionality, followed by graph-based clustering and non-linear dimensional reduction using UMAP. To mitigate the influence of doublets, DoubletFinder(15) was utilized to detect potential doublet cells. For each single-cell sample, we determined the optimal pK values and employed homotypic doublet proportion estimation. Given the variability in technologies, experiments, and human samples, we performed analyses on an organ basis and applied the Harmony(16) method to remove potential batch effects and integrate datasets from various platforms. Shared nearest neighbor clustering was based on Harmony-reduced data, which was used to generate a two-dimensional UMAP for visualization. To further annotate cell types within the identified clusters in the kidney, ureter, and bladder, we employed the Seurat FindAllMarkers function. Differential gene expression analysis was conducted within each cluster using a log fold-change threshold of 0.25, a minimum percent threshold of 0.20, and an adjusted p-value threshold of 0.05 using the Wilcox-Rank sum test. This approach provided a list of cell-type-specific genes, which were then used to manually annotate human cell types. To further explore similarities and trace biomarker expression in cells derived from the kidney, ureter, and bladder, we followed a similar Seurat pipeline and used Harmony to remove potential batch effects. A global normalization and scaling using highly variable features was applied to ensure appropriate comparisons across organs. A combined UMAP visualization was generated to assess similarities among these organs, and each protein marker was evaluated across cell types in the kidney and urinary tract. Hierarchical clustering was applied to each category of biomarkers to characterize their organ- and cell type-specific expression patterns.

**Reference**

1. Kong AT, Leprevost FV, Avtonomov DM, Mellacheruvu D, Nesvizhskii AI. MSFragger: ultrafast and comprehensive peptide identification in mass spectrometry–based proteomics. Nature methods. 2017;14(5):513-20.

2. Teo GC, Polasky DA, Yu F, Nesvizhskii AI. Fast deisotoping algorithm and its implementation in the MSFragger search engine. Journal of proteome research. 2020;20(1):498-505.

3. Yu F, Haynes SE, Teo GC, Avtonomov DM, Polasky DA, Nesvizhskii AI. Fast quantitative analysis of timsTOF PASEF data with MSFragger and IonQuant. Molecular & Cellular Proteomics. 2020;19(9):1575-85.

4. Käll L, Canterbury JD, Weston J, Noble WS, MacCoss MJ. Semi-supervised learning for peptide identification from shotgun proteomics datasets. Nature methods. 2007;4(11):923-5.

5. Nesvizhskii AI, Keller A, Kolker E, Aebersold R. A statistical model for identifying proteins by tandem mass spectrometry. Analytical chemistry. 2003;75(17):4646-58.

6. da Veiga Leprevost F, Haynes SE, Avtonomov DM, Chang H-Y, Shanmugam AK, Mellacheruvu D, et al. Philosopher: a versatile toolkit for shotgun proteomics data analysis. Nature methods. 2020;17(9):869-70.

7. Yu F, Haynes SE, Nesvizhskii AI. IonQuant enables accurate and sensitive label-free quantification with FDR-controlled match-between-runs. Molecular & Cellular Proteomics. 2021;20:100077.

8. Han X, Zhou Z, Fei L, Sun H, Wang R, Chen Y, et al. Construction of a human cell landscape at single-cell level. Nature. 2020;581(7808):303-9.

9. Wilson PC, Wu H, Kirita Y, Uchimura K, Ledru N, Rennke HG, et al. The single-cell transcriptomic landscape of early human diabetic nephropathy. Proceedings of the National Academy of Sciences. 2019;116(39):19619-25.

10. Muto Y, Wilson PC, Ledru N, Wu H, Dimke H, Waikar SS, et al. Single cell transcriptional and chromatin accessibility profiling redefine cellular heterogeneity in the adult human kidney. Nature communications. 2021;12(1):2190.

11. Muto Y, Dixon EE, Yoshimura Y, Wu H, Omachi K, Ledru N, et al. Defining cellular complexity in human autosomal dominant polycystic kidney disease by multimodal single cell analysis. Nature communications. 2022;13(1):6497.

12. Fink EE, Sona S, Tran U, Desprez P-E, Bradley M, Qiu H, et al. Single-cell and spatial mapping Identify cell types and signaling Networks in the human ureter. Developmental cell. 2022;57(15):1899-916. e6.

13. Yu Z, Liao J, Chen Y, Zou C, Zhang H, Cheng J, et al. Single-cell transcriptomic map of the human and mouse bladders. Journal of the American Society of Nephrology. 2019;30(11):2159-76.

14. Hao Y, Stuart T, Kowalski MH, Choudhary S, Hoffman P, Hartman A, et al. Dictionary learning for integrative, multimodal and scalable single-cell analysis. Nature biotechnology. 2024;42(2):293-304.

15. McGinnis CS, Murrow LM, Gartner ZJ. DoubletFinder: doublet detection in single-cell RNA sequencing data using artificial nearest neighbors. Cell systems. 2019;8(4):329-37. e4.

16. Korsunsky I, Millard N, Fan J, Slowikowski K, Zhang F, Wei K, et al. Fast, sensitive and accurate integration of single-cell data with Harmony. Nature methods. 2019;16(12):1289-96.
